# Supplementary material for: Reducing MSH4 copy number prevents meiotic crossovers between non-homologous chromosomes in Brassica napus
Source: Nat Commun. 2019 May 29;10:2354. doi: 10.1038/s41467-019-10010-9 (PMC6541637; doi:10.1038/s41467-019-10010-9)
Supplement: Supplementary file 1 — Supplementary Information [file 41467_2019_10010_MOESM1_ESM.pdf]

SUPPLEMENTARY MATERIAL

Reducing *MSH4* copy number prevents meiotic crossovers between non-homologous chromosomes in *Brassica napus*

Adrián Gonzalo[1,2], Marie-Odile Lucas[3], Catherine Charpentier[1], Greta Sandmann[1], Andrew Lloyd[1,4] and Eric Jenczewski[1,*]

[1] Institut Jean-Pierre Bourgin, INRA, AgroParisTech, CNRS, Université Paris-Saclay, 78000, Versailles, France

[2] current affiliation: Department of Cell and Developmental Biology, John Innes Centre, Norwich, NR4 7UH, United Kingdom

[3] INRA UMR1349 Institut de Génétique, Environnement et Protection des Plantes, France

[4] current affiliation: Institute of Biological, Environmental, and Rural Sciences, Aberystwyth University, Aberystwyth SY23 3EB, United Kingdom

[*] corresponding author: eric.jenczewski@inra.fr

Supplementary Table 1: ***MSH4* homologs in *Brassica* species**. Summary of the hits obtained from querying *Ath.MSH4* against *Brassica* genomes.

Organism	Genome	ID	Number of exons	Identity with query
A. thaliana		At4g17380	24	100%
B. napus	A	BnaA08g08260D	24	91.3%
B. napus	A	BnaA08g08250D	4	91.8%
B. napus	C	BnaCnng35120D	24	89.4%
B. rapa	A	Bra021052	24	91.2%
B. oleraceae	C	Bo8g048310	24	91.1%

Supplementary Table 2: Transcriptional behaviour of wt and mutant *BnaA.MSH4* and *BnaC.MSH4*. Results of *MSH4* transcript analysis results in euploids. Expression results are expressed as normalized fold change ($2^{-\Delta\Delta Cq}$). Pyrosequencing results are expresed in form of percentage normalized with genomic DNA.

Genotype	Plants analyzed	Expression	Expression SD	C-contribution	A-contribution
Darmor	1	nd	nd	83%	17%
Yudal	1	nd	nd	72%	28%
Tanto	2	0.56	0.08	78%	22%
$A^+A^+C^+C^+$	3	1.00	0.06	79%	21%
$A^1A^1 C^+C^+$	3	0.62	0.26	89%	11%
$A^+A^+C^1C^1$	3	0.36	0.20	72%	28%
$A^+A^1C^1C^1$	3	0.57	0.18	81%	19%
$A^1A^1C^1C^1$	3	0.51	0.15	78%	22%
A^+C^+	3	1.00	0.13	79%	21%
A^1C^+	3	1.94	1.14	79%	21%
A^+C^1	1	1.52		75%	25%
$A+C^2$	1	0.49		76%	24%
A^2C^1	1	1.17		nd	nd

Supplementary Table 3: All the primers used in this study are listed along with their Tm.

Name	Sequence 5'-3'	Tm (ºC)
Q_UBC21F1	CCTCTGCAGCCTCCTCAAGT	62
Q_UBC21R1	GTGTACATGTGTGCCATTGA	62
Q_MSH4F1	GATTGTCACCAACCACTCACTCAC	62
Q_MSH4R1	CCTCTGCAGCCTCCTCAAGT	62
PS_MSH41F	TGCTCAGATTGGCTGCTATGT	62
PS_MSH4S	CAACCACACGCATAGT	62
PS_MSH4R	TTCTTGTGAATATGCGGTCAAC	62
T_MSH4AF1	TTGACCAAAAAATCATCATCGG	63
T_MSH4AR1	TGCTATGCTGTAATGAATCCAGAT	63
T_MSH4CF1	TTGTCCAAAAAATGTCCTCATCGA	63
T_MSH4CR1	CTCAAGACACAAGGATGTATTACGA	63
Amsh4-57F	TGATGGCAAATGAACTTTCC	54.5
Amsh4-57R	GCGAGTGGGATGAAGTC	54.5
Cmsh4-71F	GACGGGTTAGCAATGCCATG	59.4
Cmsh4-71R	CATTCGGGTAGATGGTTGCC	59.4
Cmsh4-44F	ATCGCTGAAAGGTACTTGAC	52
Cmsh4-44R	CAGAACCCATGCGACT	52

Supplementary Table 4: List of restriction markers for identification of *msh4* alleles

Allele	Primer pair	Enzime	Size of restriction fragments (bp)	
			**Wt**	**mutant**
BnaA.msh4-1	Amsh4-57F<br>Amsh4-57R	HpaII	139+19	158
BnaC.msh4-1	Cmsh4-71F<br>Cmsh4-71R	NcoI	196+16	212
BnaC.msh4-2	Cmsh4-44F<br>Cmsh4-44R	HphI	177+32	209

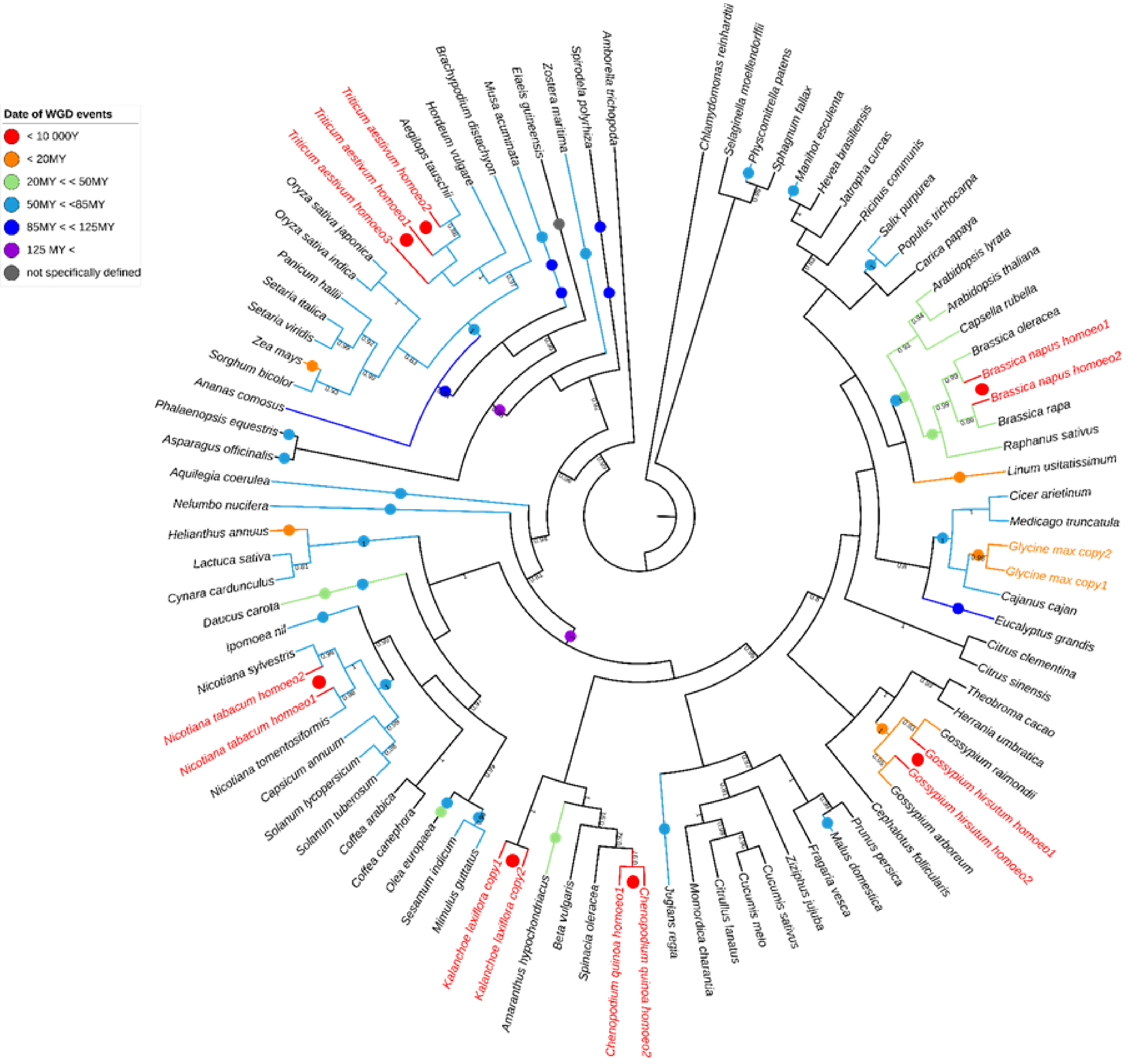

Supplementary Figure 1: Phylogeny of MSH5 in angiosperms

A Maximum Likelihood tree based on amino acid sequences is provided. For the sake of clarity, species names are indicated instead of gene names. Branch support is given as Shimodaira-Hasegawa-like Likelihood Ratio Test (aLRT SH-like).

Coloured disks superimposed along the branches of the trees give the age range for past WGDs. Full-length duplicates and recent duplicated with one fractionated copies are written with the color that corresponds to the age of the WGD (i.e. red: <10,000 years; orange: <20MY; light blue: 50MY< < 85MY).

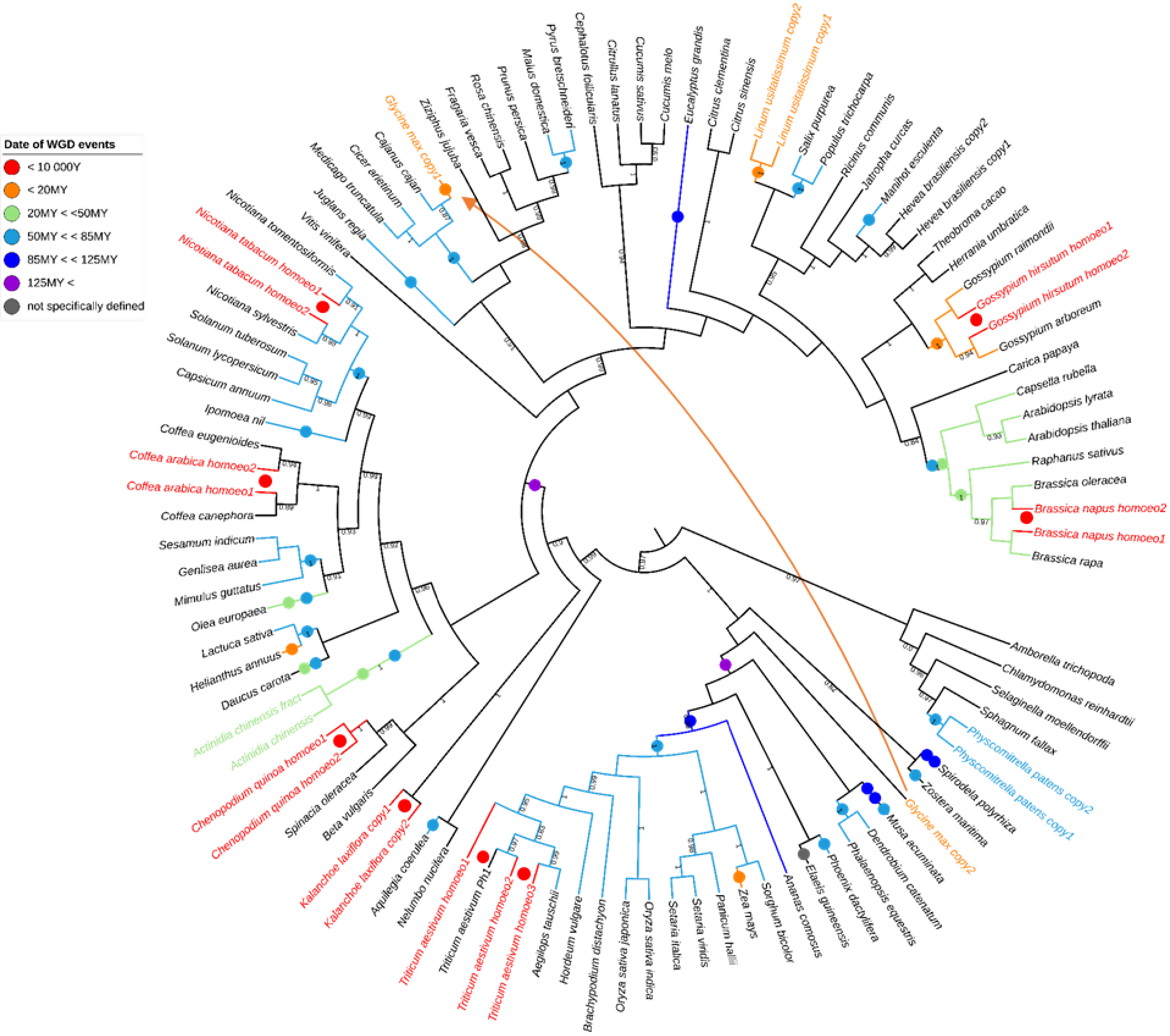

Supplementary Figure 2: Phylogeny of ZIP4 in angiosperms

A Maximum Likelihood tree based on amino acid sequences is provided. For the sake of clarity, species names are indicated instead of gene names. Branch support is given as Shimodaira-Hasegawa-like Likelihood Ratio Test (aLRT SH-like).

Coloured disks superimposed along the branches of the trees give the age range for past WGDs. The extra copy of ZIP4 found in bread wheat (*T.aestivum Ph1*) is indicated in black because it originated from a Small Scale Duplications. Full-length duplicates and recent duplicated with one fractionated copies are written with the color that corresponds to the age of the WGD (i.e. red: <10,000 years; orange: <20MY; light green: 25MY< < 50MY). The connector superimposed over the phylogeny associates the full-length copy of *ZIP4* with a fractionated copy that originated from the same specific WGD (e.g. in *Glycine max*).

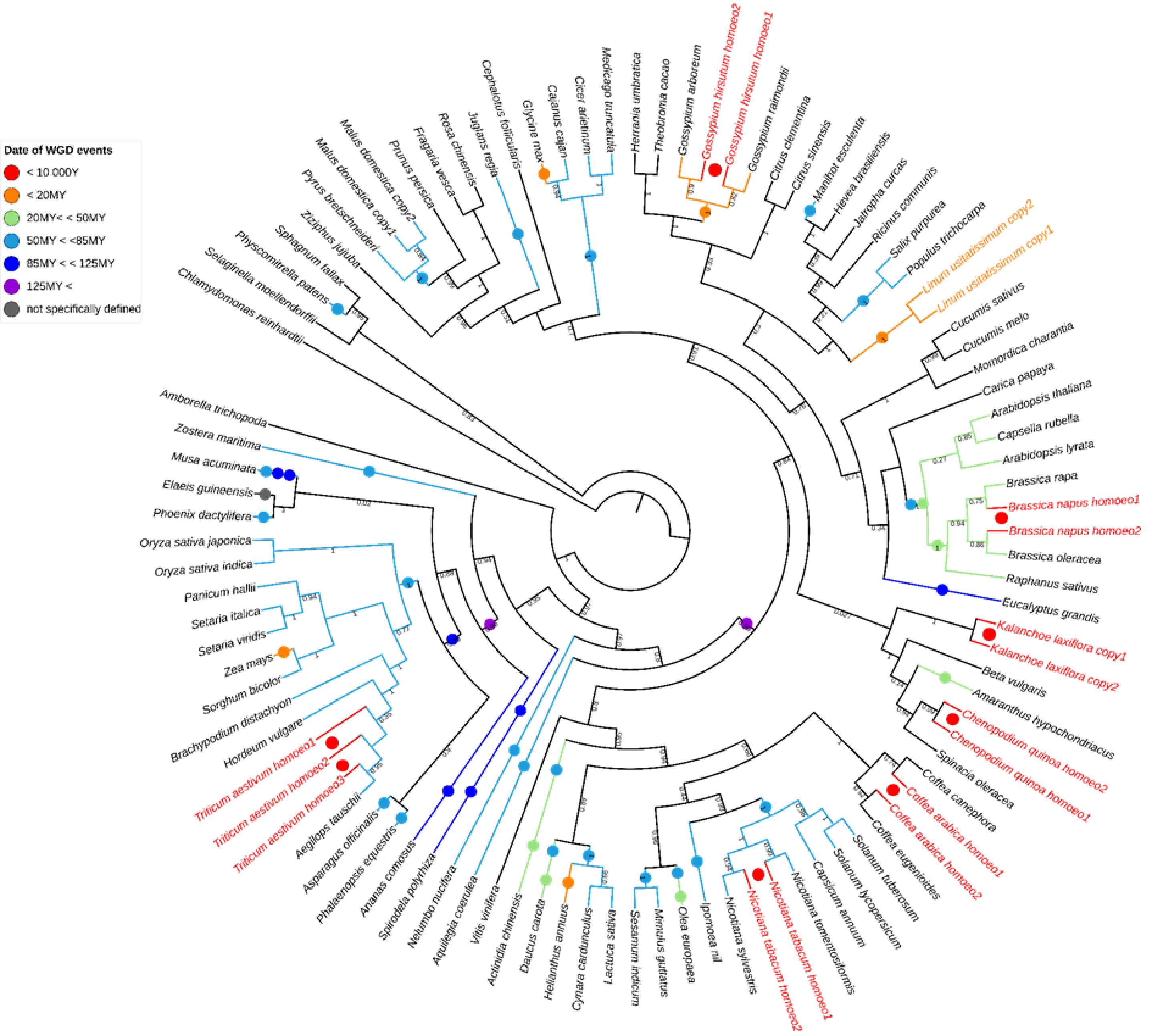

Supplementary Figure 3: Phylogeny of MER3 in angiosperms

A Maximum Likelihood tree based on amino acid sequences is provided. For the sake of clarity, species names are indicated instead of gene names. Branch support is given as Shimodaira-Hasegawa-like Likelihood Ratio Test (aLRT SH-like).

Coloured disks superimposed along the branches of the trees give the age range for past WGDs. Full-length duplicates are written with the color that corresponds to the age of the WGD (i.e. red: <10,000 years; orange: <20MY for *L. usitatissimum*).

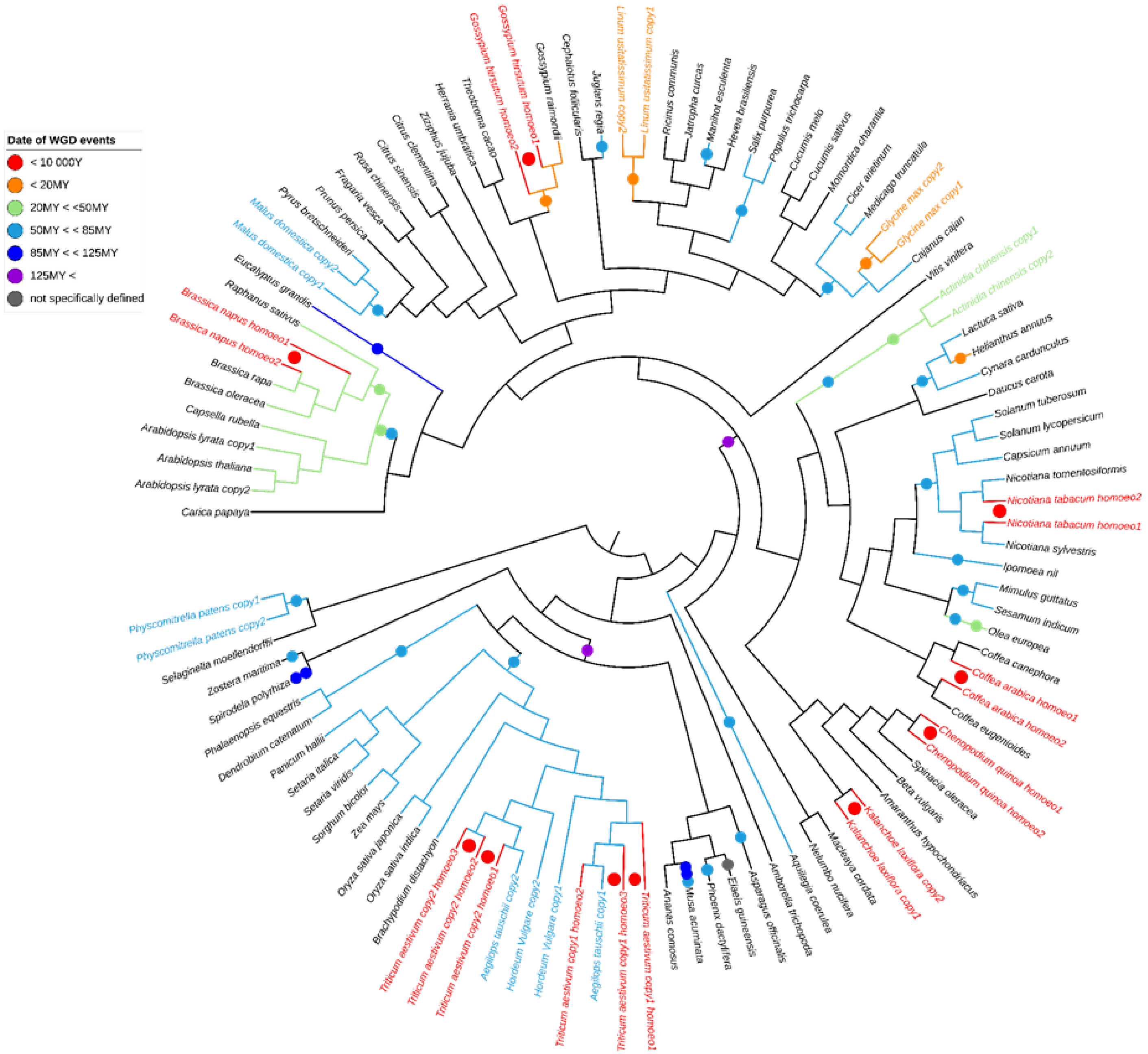

Supplementary Figure 4: Phylogeny of SHOC1 in angiosperms

A Maximum Likelihood tree based on amino acid sequences is provided. For the sake of clarity, species names are indicated instead of gene names. Branch support is given as Shimodaira-Hasegawa-like Likelihood Ratio Test (aLRT SH-like).

Coloured disks superimposed along the branches of the trees give the age range for past WGDs. Full-length duplicates are written with the color that corresponds to the age of the WGD (i.e. red: <10,000 years; orange: <20MY; light green: 20MY< < 50MY; light blue: 50MY< < 85MY).

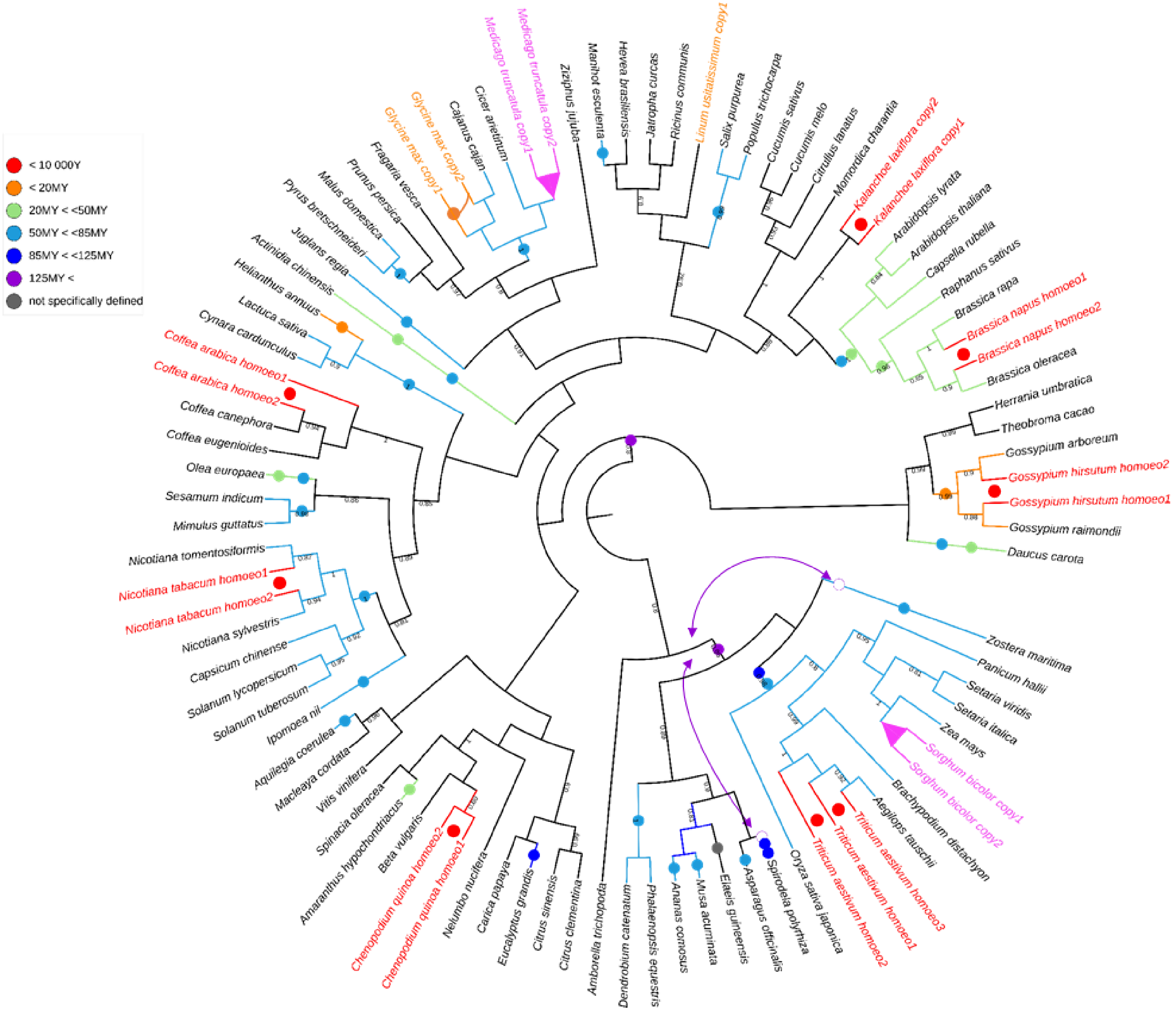

Supplementary Figure 5: Phylogeny of PTD in angiosperms

A Maximum Likelihood tree based on amino acid sequences is provided. For the sake of clarity, species names are indicated instead of gene names. Branch support is given as Shimodaira-Hasegawa-like Likelihood Ratio Test (aLRT SH-like).

Coloured disks superimposed along the branches of the trees give the age range for past WGDs. The pink triangles (and associated copies) represents tandem duplicates. Full-length duplicates are written with the color that corresponds to the age of the WGD (i.e. red: <10,000 years; orange: <20MY).

The connectors superimposed over the phylogenies are used to associate the duplicates originating from a specific WGD but that scattered around the phylogeny (*G. max*). Connectors associated with open circle along a branch are used to correct the misplacement of some species with respect to some past WGDs; i.e. to show that *Zostera marit*ima and *Spirodela polyrhiza* did not experience the WGD that is common to all other monocots.

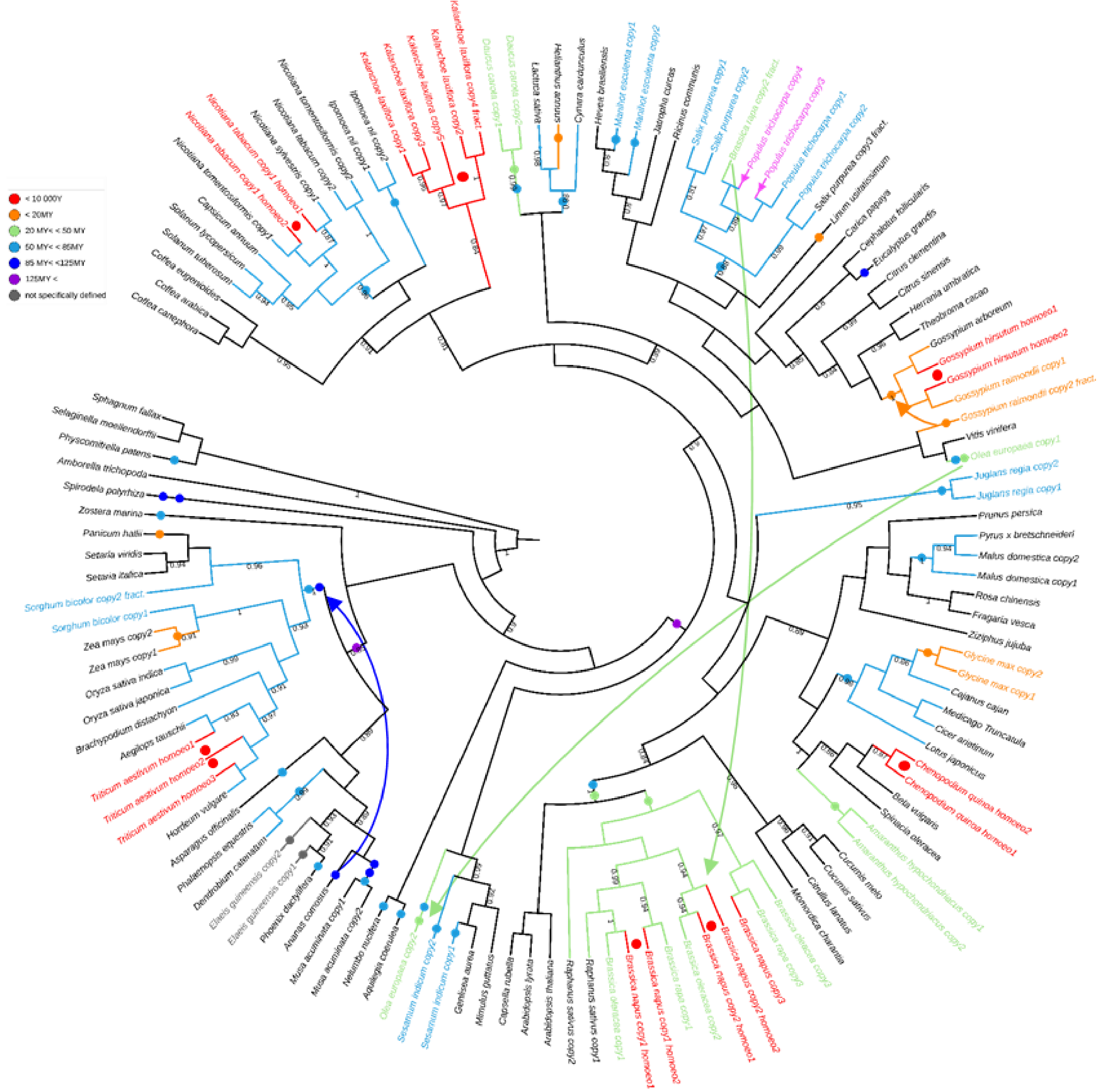

Supplementary Figure 6: Phylogeny of HEI10 in angiosperms

A Maximum Likelihood tree based on amino acid sequences is provided. For the sake of clarity, species names are indicated instead of gene names. Branch support is given as Shimodaira-Hasegawa-like Likelihood Ratio Test (aLRT SH-like).

The pink triangle (and associated copies) represents tandem duplicates. Full-length duplicates and recent duplicated with one fractionated copies are written with the color that corresponds to the age of the WGD (i.e. red: <10,000 years; orange: <20MY; light blue: 50MY< < 85MY).

The connectors superimposed over the phylogenies are used to associate the duplicates that originated from a specific WGD but scattered around the phylogeny: e.g. *Olea europea, Gossypium raimondii* or *Brassica rapa*. Connectors associated with open circle along a branch are also used to correct the misplacement of some species with respect to some past WGDs; i.e. to show that *Ananas comosus* share a common WGD with all grasses

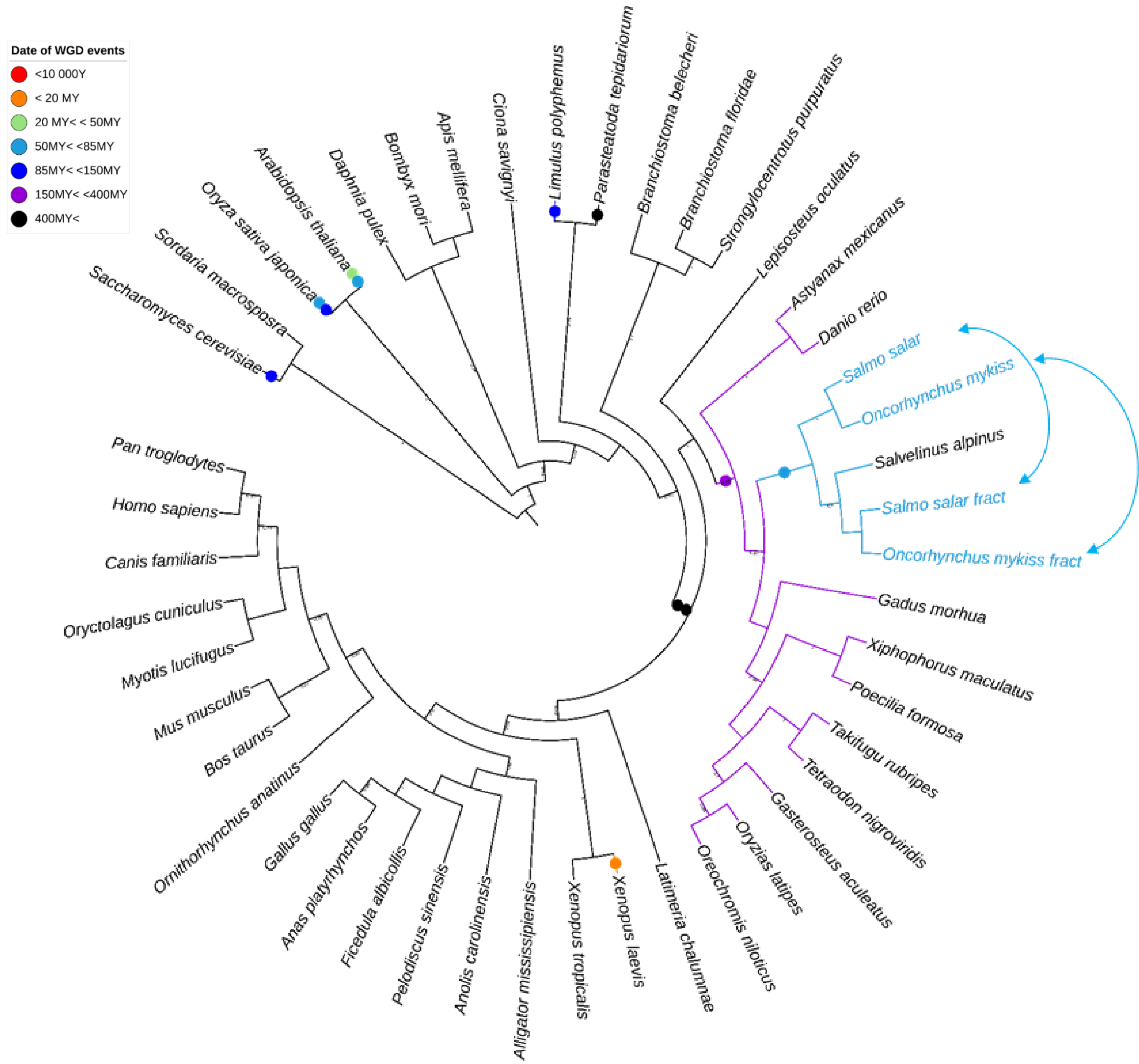

Supplementary Figure 7: Phylogenies of MSH4 in animals

Maximum Likelihood trees based on MSH4 amino acid sequences is provided. For the sake of clarity, species names are indicated instead of gene names. Branch support is given as Shimodaira-Hasegawa-like Likelihood Ratio Test (aLRT SH-like).

Coloured disks superimposed along the branches of the trees give the age range for past WGDs. Note that this Figure does not reflect all of the WGDs that contributed to the evolution of animals. The reason is that many of the additional unrepresented WGD events were detected using somatic transcriptome data[1] from which MSH4 is excluded.

The connectors superimposed over the phylogenies are used to associate the duplicates (usually a full-length copy and a fractionated one) originating from a specific WGD but that scattered around the phylogeny (because the fractionated copy is highly divergent).

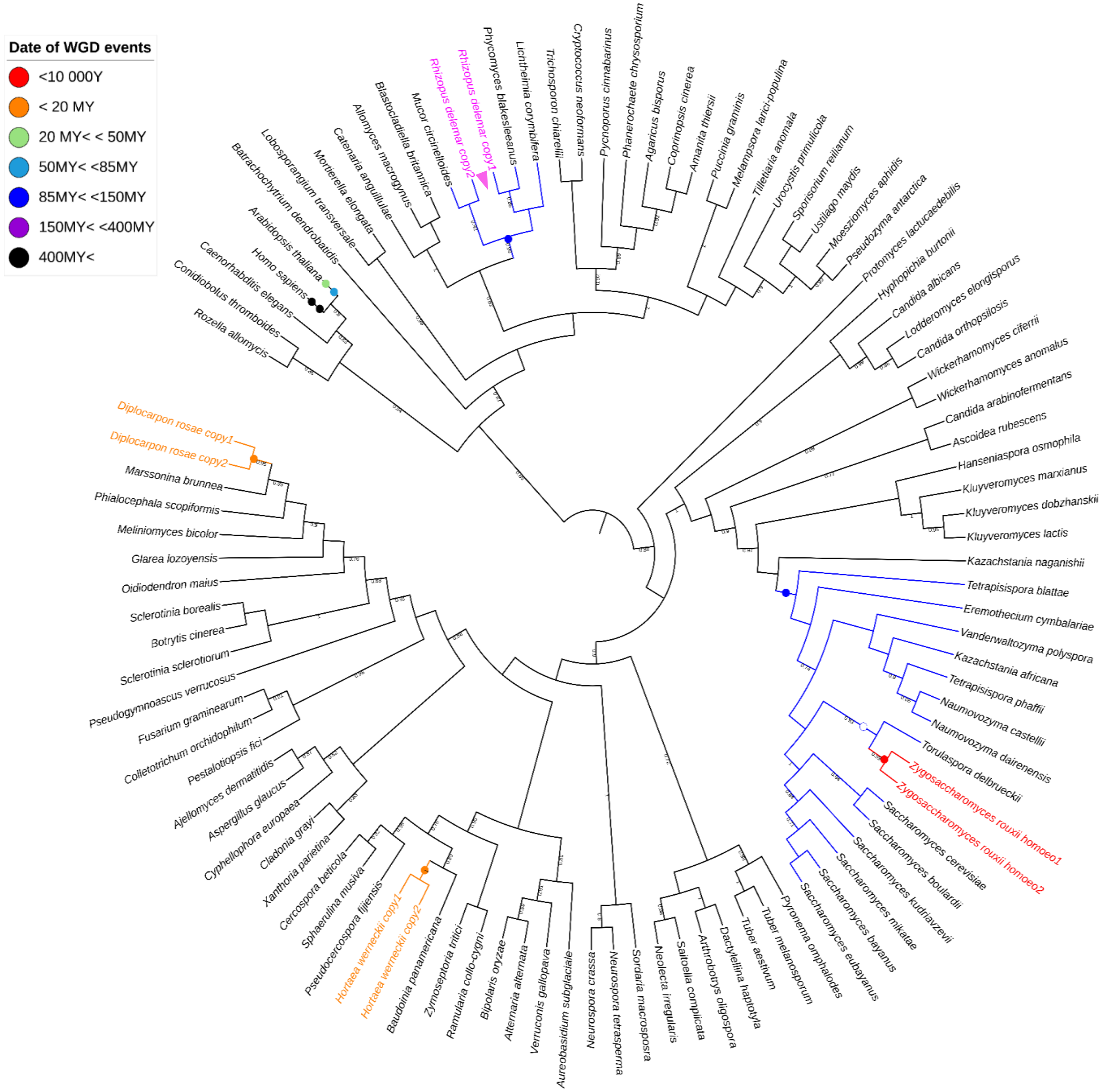

Supplementary Figure 8: Phylogenies of MSH4 in animals and fungi

Maximum Likelihood trees based on MSH4 amino acid sequences is provided. For the sake of clarity, species names are indicated instead of gene names. Branch support is given as Shimodaira-Hasegawa-like Likelihood Ratio Test (aLRT SH-like).

Coloured disks superimposed along the branches of the trees give the age range for past WGDs. The ppen disk indicates a burst of gene duplication that have not been formally associated with a WGD event[2]. The pink triangle and associated *Rhizopus delemar* copie represents tandem duplicates. Regarding the most recent WGD (i.e. red: <10,000 years), only allopolyploids are represented because there is no way to account for the presence of *MSH4* “duplicates” in extant autopolyploids, which truly correspond to (multiple) real alleles.

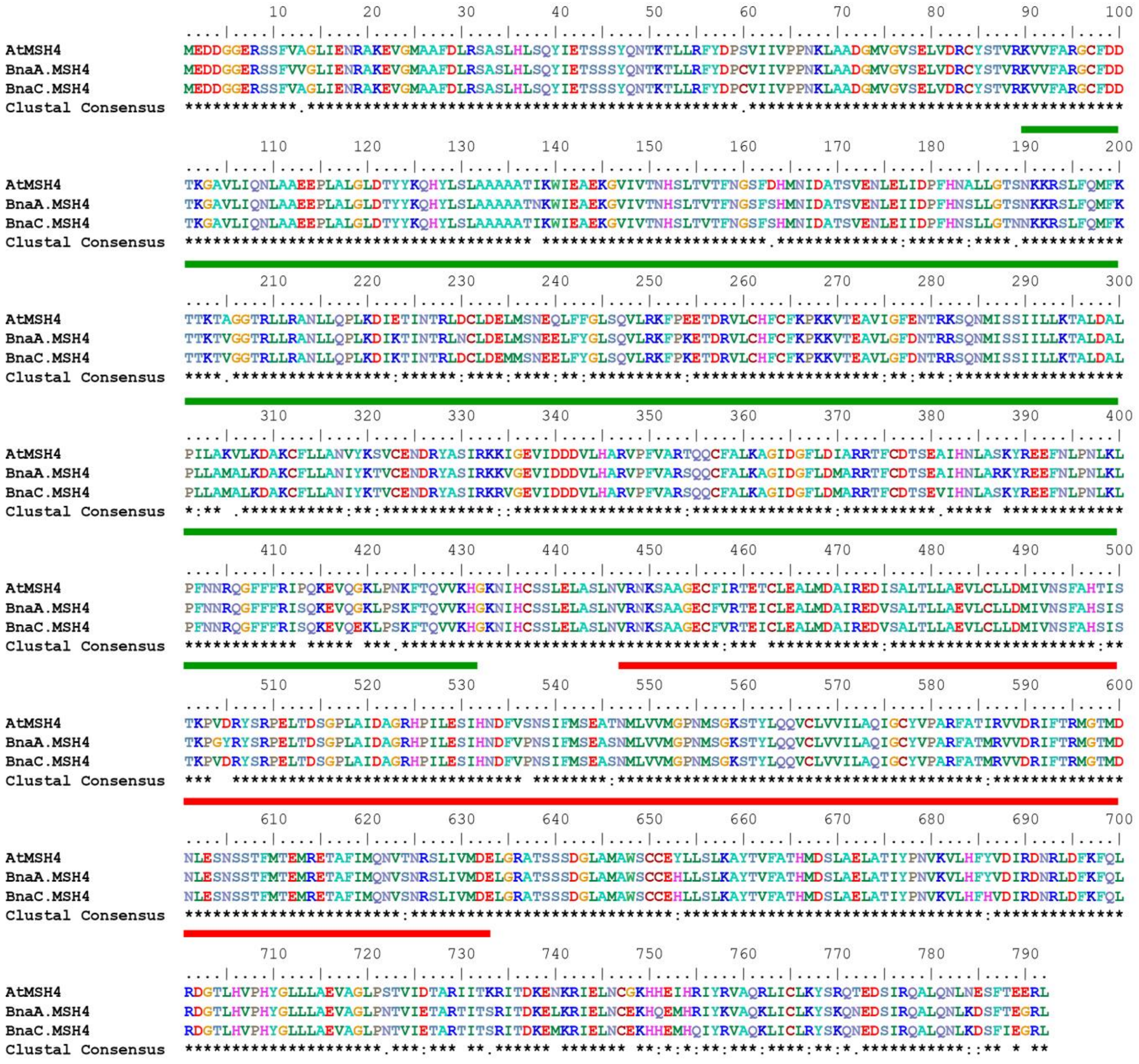

Supplementary Figure 9. MSH4 proteins in Brassica

Amino acid sequence alignment of BnaA.MSH4 and BnaC.MSH4 with their *A. thaliana* ortholog (AtMSH4).

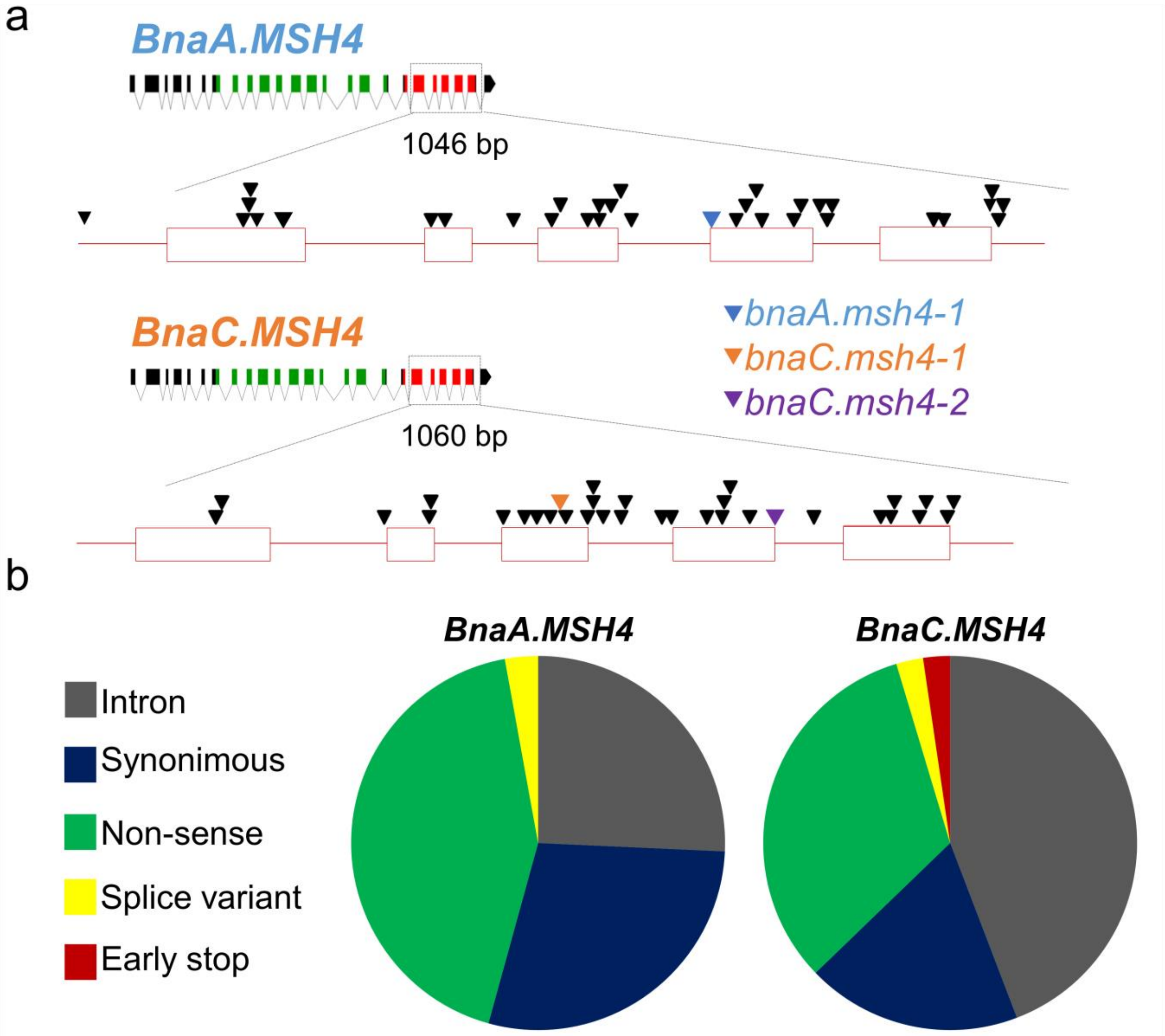

Supplementary Figure 10: Overview of the *msh4* mutations identified by TILLING in *Brassica napus*. (a) Schematic representation of *BnaA.MSH4* and *BnaC.MSH4* exon-intron structure identifying the MutSd (green) and MutSac (red) domains (as predicted using the SMART online blast tool SMART). A zoom on the region of the MutSac domain where mutations were searched for is provided for the two genes. An arrowhead marks the position of all identified mutations, including *bnaA.msh4-1* (light blue), *bnaC.msh4-1* (magenta) and *BnaC.msh4-2* (orange). (b) Distribution of mutations in *BnaA.MSH4* and *BnaC.MSH4*, respectively, according to their type.

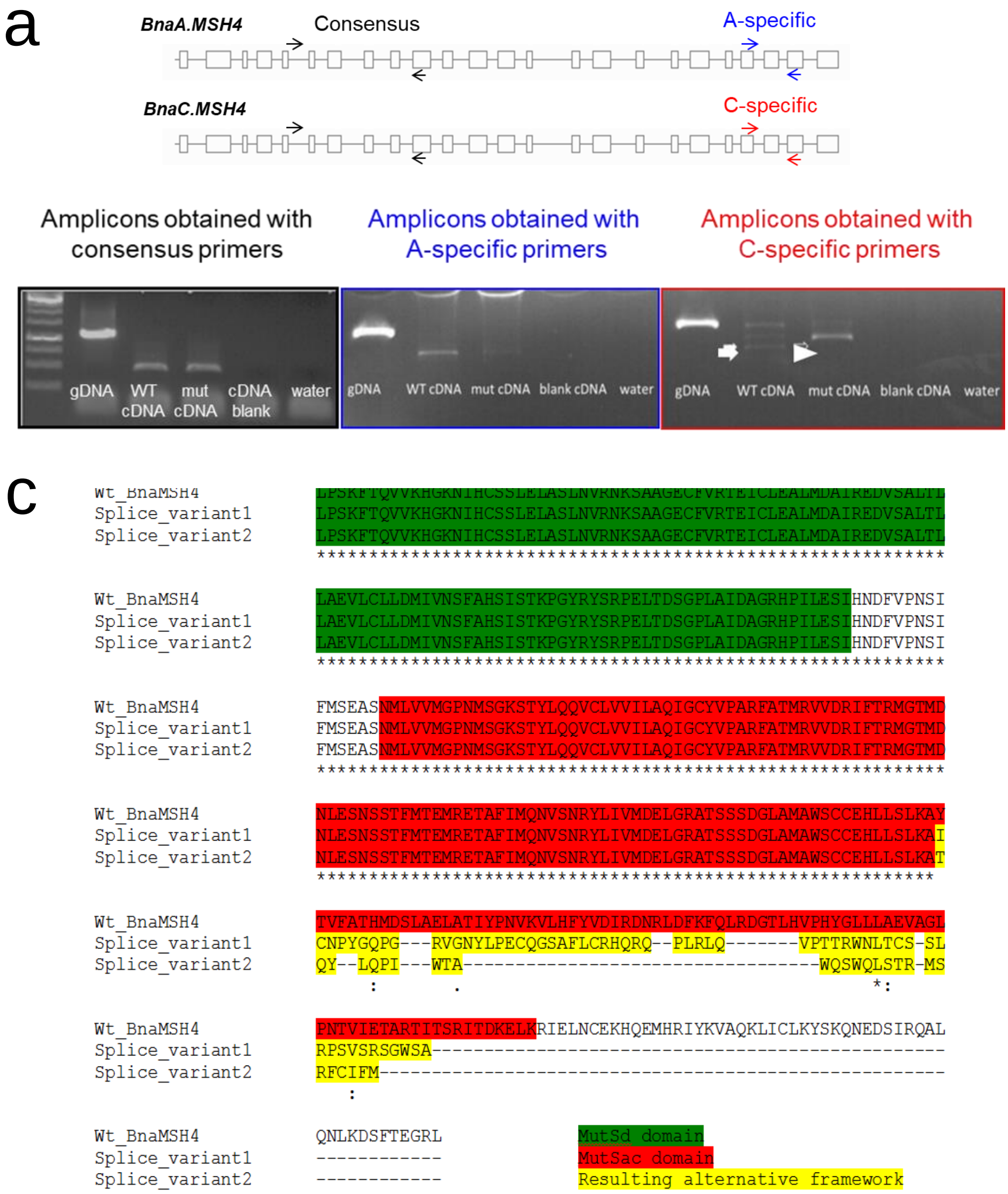

Supplementary Figure 11: Splicing variation in *bnaA.msh4-1* and *bnaC.msh4-2*.

(a) shows a schematic representation of the position of primers used to assess the splicing pattern associated with *bnaA.msh4-1* and *bnaC.msh4-2* mutations, respectively. Black arrows represent the consensus primers that we used to test for the presence of genomic DNA (gDNA). Blue and red arrows represent the pairs of A- and C-copy specific primers, respectively. Amplicon specificity was verified by Sanger sequencing (not shown here).

(b) shows the resulting PCR amplicons obtained using genomic DNA (gDNA), cDNA obtained from meiotic buds in Tanto (WT cDNA) and the double mutant $A^1A^1C^2C^2$ (mut cDNA). Blank cDNA (no template for RT) and water are used as negative controls for PCR reactions. One band was produced by *bnaA.msh4-1*, which is very similar in size than that of the wt. Sanger sequencing revealed that this band actually contains two amplicons that show 1 and 16 short deletions compared to the wt sequence, respectively (not shown). By contrast, three bands were obtained using C-copy specific primers and wt cDNA. Only two of these fragments were amplified using mut cDNA, the missing band (arrowhead) corresponding to the correctly spliced mRNA (arrow).

(c) shows the alignment of the predicted amino acid sequences encoded by the two aberrant splice variants produced by *bnaA.msh4-1* along with the WT AA sequence. The two spliced variants lead to frameshifts, each followed by premature stop codons.

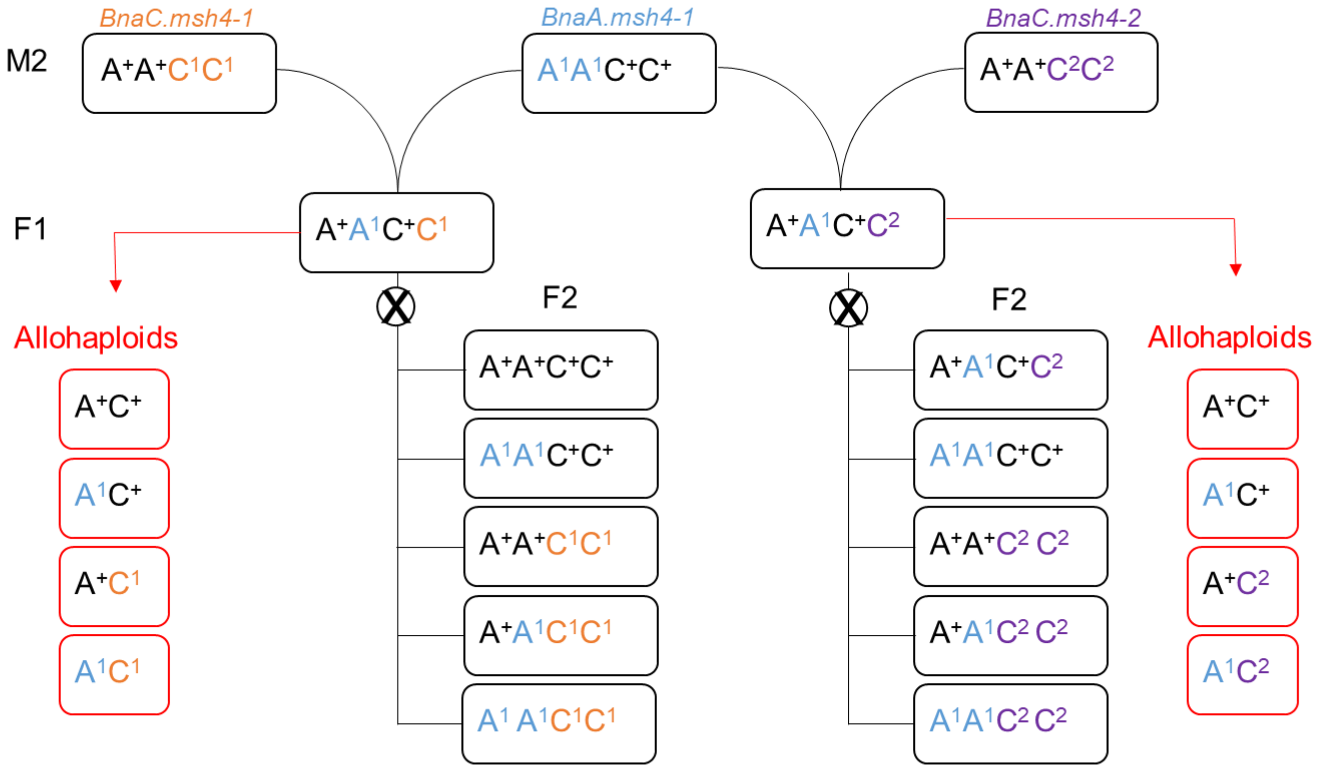

Supplementary Figure 12: Genealogy of the plants used to evaluate the consequences of *MSH4* duplicate loss in *B. napus*.

Plants homozygous for *bnaA.msh4-1*, *bnaC.msh4-1* and *bnaC.msh4-2* were selected within the corresponding M2 families (from the RapTill population) and crossed to produce two different F1 hybrids. These F1s were self-fertilized to produce F2 progenies among which, plants containing varied number and assortments of Wild Type (A^+ or C^+) and mutant *msh4* alleles (A^1, C^1 or C^2) were selected. The red lines indicate isolation of allohaploid plants through microspore culture.

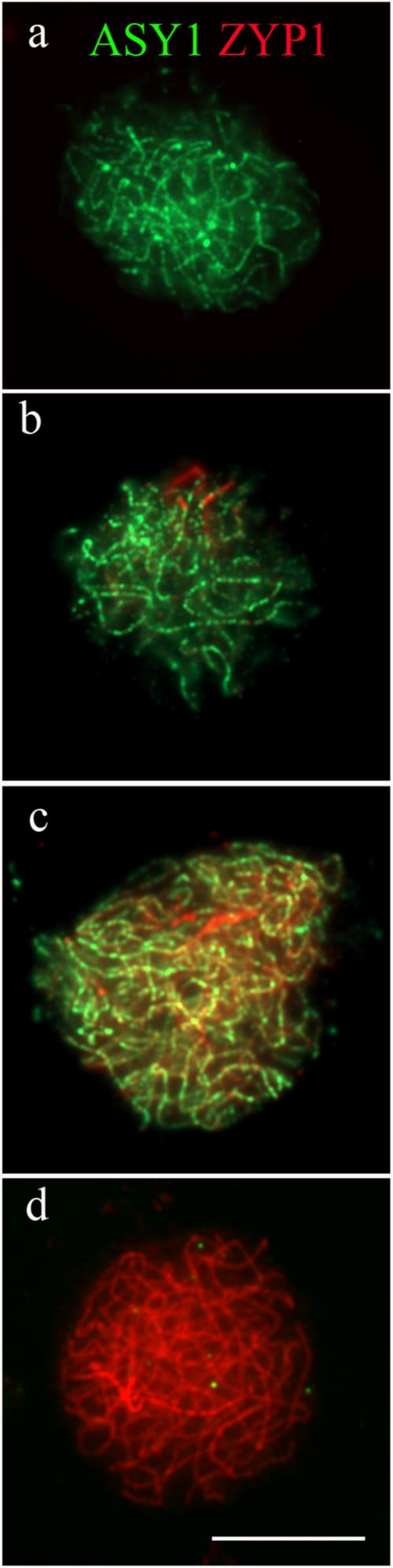

Supplementary Figure 13: Normal Synaptonemal Complex formation during meiosis in *MSH4*-deficient $A^1A^1C^1C^1$ *B.napus* plant.

Immunolocalization of AtASY1 (green; axial/lateral elements) and AtZYP1 (red; central element) antibodies to spread *B.napus* Pollen Mother Cells. (a) late leptotene showing the axial element of every chromosome marked by ASY1. (b) zygotene showing incipient synapsis marked by the first ZYP1 tracts. (c) late/mid zygotene showing the progression of synapsis, which is eventually achieved at pachytene when the ZYP1 signal is continuous (d).
Bar scale 10 µm.

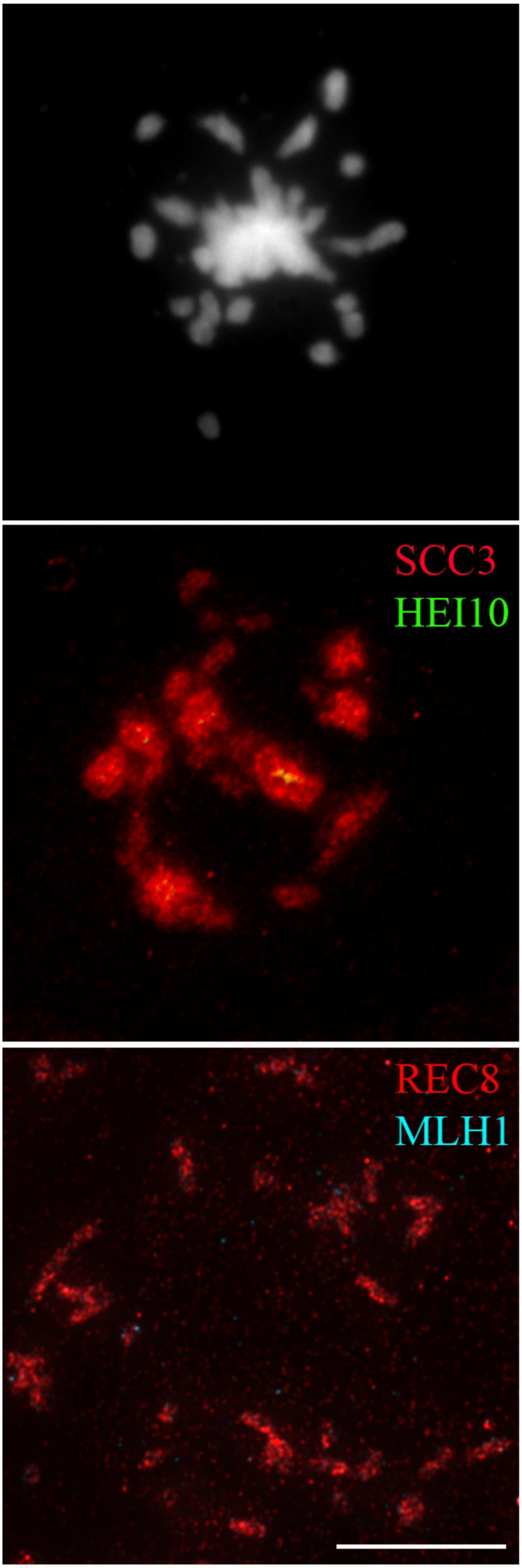

Supplementary Figure 14: Defective crossover formation in *MSH4*-deficient $A^1A^1C^2C^2$ *B.napus* plant.

The upper pictures show DAPI spreads of metaphase I. The middle pictures show dual immunolocalization of SCC3 and HEI10 at diakinesis stage. The lower pictures show dual immunolocalization of REC8 and MLH1. Bar scale 10 µm.

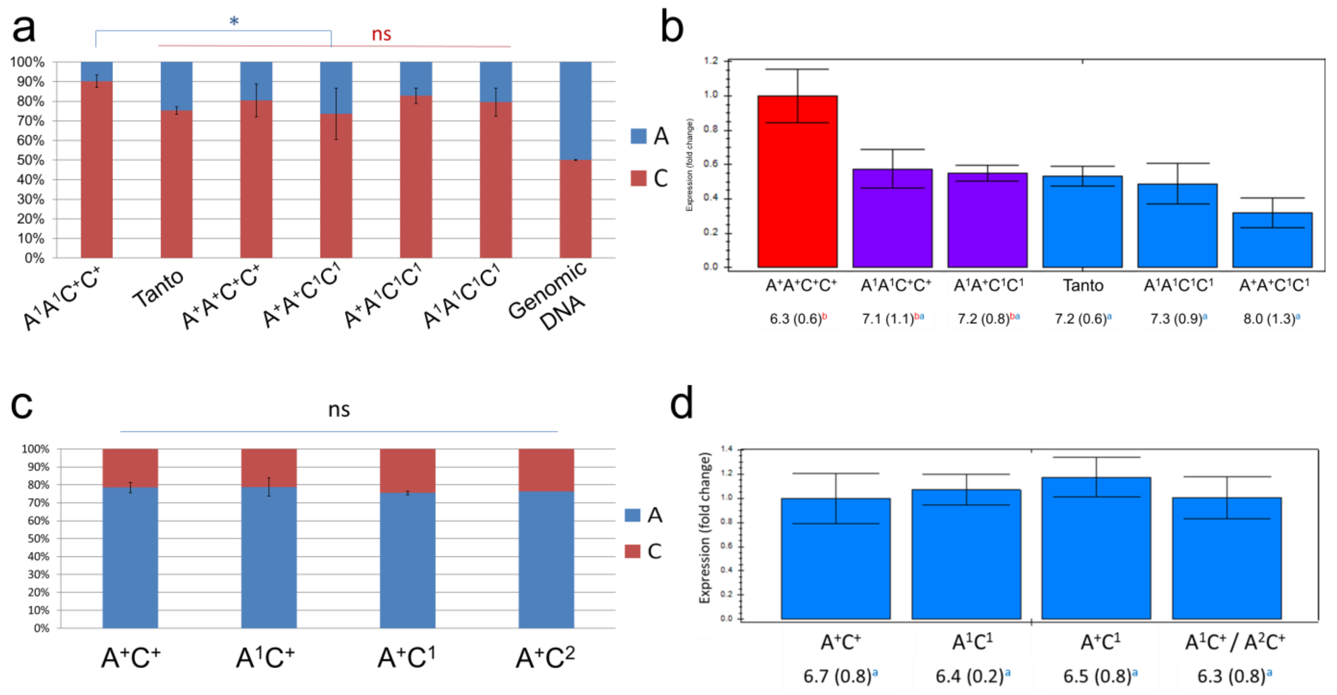

Supplementary Figure 15: Transcriptional robustness against loss of *MSH4* functional copies.

(**a** and **c**) Relative contribution of *BnaA.MSH4* and *BnaC.MSH4* to total *MSH4* transcripts evaluated by pyrosequencing in euploids (a) and allohaploids (c). Proportions were normalized based on the results obtained with genomic DNA. Error bars represent SD. The Marascuillo procedure was used to compare the *BnaA.MSH4* / *BnaC.MSH4* proportions between genotypes. * marks the statistically significant difference (absolute difference: 0.16; critical value: 0.099) while ns stands for non-significant variation.
(**b** and **d**) Quantification of the summed expression of *BnaA.MSH4* and *BnaC.MSH4* by real-time PCR, expressed as the normalized fold change ($2^{-\Delta\Delta Cq}$) in the target sample relative to the $A^+A^+C^+C^+$ genotype (for euploids, b) or to the A^+C^+ genotype (for allohaploids, d). Error bars represent SD.
Post-ANOVA Tukey's tests were carried out to compare the difference in threshold cycle between *MSH4* and *UBC21* (i.e. the dependent variable) between genotypes. Means with the same letter are not significantly different (alpha: 0.05 ; Df Error: 56; Critical Value of Studentized Range: 4.172863). For the ANOVA, Genotype effect: Pr(>F)=0.001 (df:5) and Plate effect: Pr(>F): 1.575e-05 *** (df:1); see Methods.

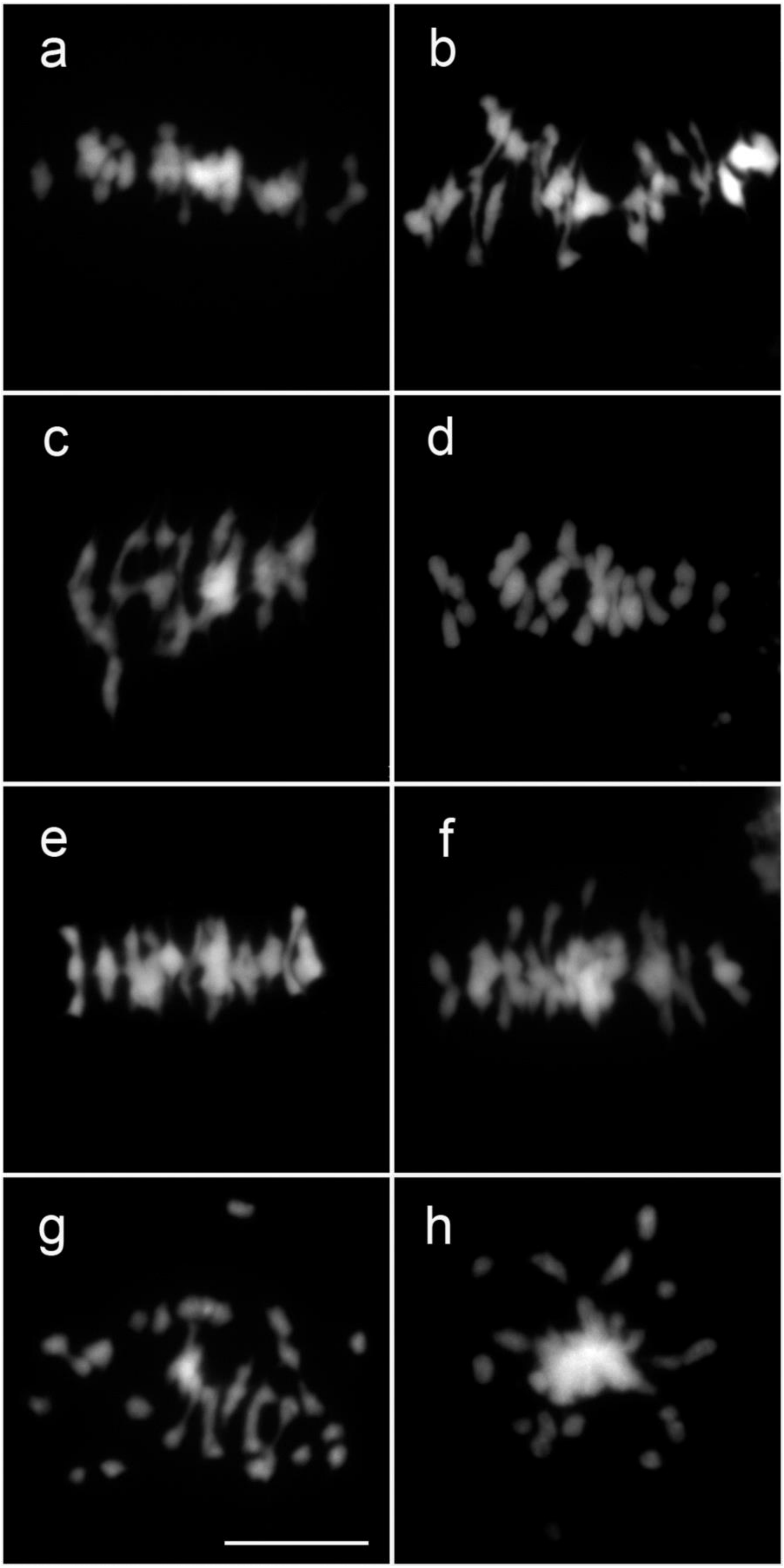

Supplementary Figure 16: Meiotic regularity in *Brassica napus* plants combining wild type (A^+, C^+) and mutant (A^1, C^1, C^2) alleles of *MSH4*.

DAPI spreads of metaphase I obtained from Tanto (a), $A^+A^+C^+C^+$ (b), $A^1A^2C^+C^+$(c), $A^+A^+C^1C^1$ (d), $A^+A^+C^2C^2$ (e), $A^+A^1C^1C^1$ (f), $A^1A^1C^1C^1$ (g), $A^1A^1C^2C^2$ (h). Bar scale 10 µm.

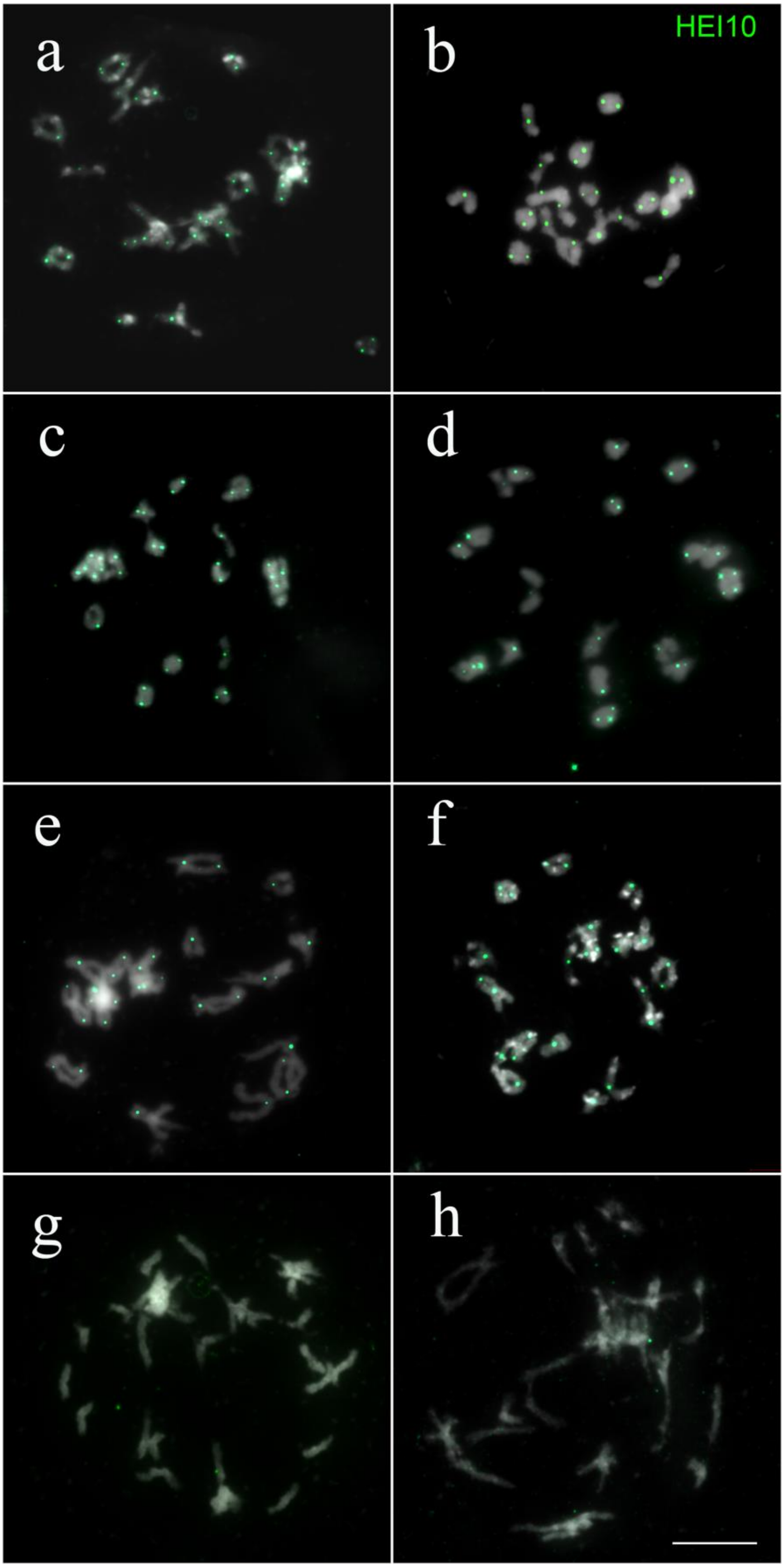

Supplementary Figure 17: HEI10-dependent crossovers in *Brassica napus* plants combining wild type (A^{+}, C^{+}) and mutant (A^{1}, C^{1}, C^{2}) alleles of *MSH4*.
HEI10 immunolocalization pictures in Tanto (**a**), $A^{+}A^{+}C^{+}C^{+}$ (**b**), $A^{1}A^{1}C^{+}C^{+}$(**c**), $A^{+}A^{+}C^{2}C^{2}$ (**d**), $A^{+}A^{+}C^{1}C^{1}$ (**e**), $A^{+}A^{1}C^{1}C^{1}$(**f**), $A^{1}A^{1}C^{1}C^{1}$(**g**) and $A^{2}A^{2}C^{1}C^{1}$ (**h**). Bar scale 10 µm.

Supplementary references

1. Li, Z. et al. Multiple large-scale gene and genome duplications during the evolution of hexapods. Proc. Natl. Acad. Sci. U. S. A. 115, 4713–4718 (2018).

2. Marcet-Houben, M. & Gabaldón, T. Beyond the Whole-Genome Duplication: Phylogenetic Evidence for an Ancient Interspecies Hybridization in the Baker’s Yeast Lineage. PLoS Biol. 13, e1002220 (2015).
