# Supplementary material for: Reducing MSH4 copy number prevents meiotic crossovers between non-homologous chromosomes in Brassica napus
Source: Nat Commun. 2019 May 29;10:2354. doi: 10.1038/s41467-019-10010-9 (PMC6541637; doi:10.1038/s41467-019-10010-9)
Supplement: Supplementary file 3 — Description of Additional Supplementary Files [file 41467_2019_10010_MOESM3_ESM.pdf]

Description of Additional Supplementary Files

File Name: Supplementary Data 1
Description: this file provides sources for the amino acid sequences used to build the phylogenies presented in Figure1, Supplementary Figure1 and Supplementary Figure2.

File Name: Supplementary Data 2
Description: This files provides the raw data we used to prepare the different figures.
