# Supplementary material for: Reducing MSH4 copy number prevents meiotic crossovers between non-homologous chromosomes in Brassica napus
Source: Nat Commun. 2019 May 29;10:2354. doi: 10.1038/s41467-019-10010-9 (PMC6541637; doi:10.1038/s41467-019-10010-9)
Supplement: Supplementary file 6 — Reporting Summary [file 41467_2019_10010_MOESM6_ESM.pdf]

nature research

Corresponding author(s): DBPR NCOMMS-18-35268A

Last updated by author(s): Apr 3, 2019

Reporting Summary

Nature Research wishes to improve the reproducibility of the work that we publish. This form provides structure for consistency and transparency in reporting. For further information on Nature Research policies, see Authors & Referees and the Editorial Policy Checklist.

Statistics

For all statistical analyses, confirm that the following items are present in the figure legend, table legend, main text, or Methods section.

n/a	Confirmed	
☐	☒	The exact sample size (*n*) for each experimental group/condition, given as a discrete number and unit of measurement
☐	☒	A statement on whether measurements were taken from distinct samples or whether the same sample was measured repeatedly
☐	☒	The statistical test(s) used AND whether they are one- or two-sided<br>*Only common tests should be described solely by name; describe more complex techniques in the Methods section.*
☐	☒	A description of all covariates tested
☐	☒	A description of any assumptions or corrections, such as tests of normality and adjustment for multiple comparisons
☐	☒	A full description of the statistical parameters including central tendency (e.g. means) or other basic estimates (e.g. regression coefficient) AND variation (e.g. standard deviation) or associated estimates of uncertainty (e.g. confidence intervals)
☐	☒	For null hypothesis testing, the test statistic (e.g. *F*, *t*, *r*) with confidence intervals, effect sizes, degrees of freedom and *P* value noted<br>*Give P values as exact values whenever suitable.*
☒	☐	For Bayesian analysis, information on the choice of priors and Markov chain Monte Carlo settings
☒	☐	For hierarchical and complex designs, identification of the appropriate level for tests and full reporting of outcomes
☒	☐	Estimates of effect sizes (e.g. Cohen's *d*, Pearson's *r*), indicating how they were calculated

Our web collection on statistics for biologists contains articles on many of the points above.

Software and code

Policy information about availability of computer code

Data collection	As described in Methods, microscopy images were obtained using the ZEISS ZEN software package (ZEISS, version 2011). Data collection for qPCR used Bio-Rad CFX Manager (version 3.1).
Data analysis	As described in Methods, microscopy images were analysed using the ZEISS ZEN software package (ZEISS, version 2011). As stated in Methods section, statistical analyses were performed using Prism GraphPad (version 5.04) and Rstudio (version 1.0.153). Graph production was performed using both Microsoft Excel and GraphPad. Calculation of qPCR values from raw data was performed using Microsoft Excel.

For manuscripts utilizing custom algorithms or software that are central to the research but not yet described in published literature, software must be made available to editors/reviewers. We strongly encourage code deposition in a community repository (e.g. GitHub). See the Nature Research guidelines for submitting code & software for further information.

Data

Policy information about availability of data

All manuscripts must include a data availability statement. This statement should provide the following information, where applicable:

- Accession codes, unique identifiers, or web links for publicly available datasets
- A list of figures that have associated raw data
- A description of any restrictions on data availability

Raw data used to produce the graphs and figures are provided in Supplementary data 2. Accession numbers of (or links to) sequences used for Figure 1, Supplementary Figure1 and Supplementary Figure 2 are provided in Supplementary data 1. The other data that support the findings of this study are available from the corresponding author upon reasonable request. We declare no restriction on data availability.

Field-specific reporting

Please select the one below that is the best fit for your research. If you are not sure, read the appropriate sections before making your selection.

☒ Life sciences ☐ Behavioural & social sciences ☐ Ecological, evolutionary & environmental sciences

For a reference copy of the document with all sections, see nature.com/documents/nr-reporting-summary-flat.pdf

Life sciences study design

All studies must disclose on these points even when the disclosure is negative.

Sample size	The sample size obtained was not determined by statistical methods. For plant material (i.e. biological replicates), sample size reflects directly the availability of the plants we were able to produce. For instance, the number allohaploid plants analysed for each genotype depended on the number of individuals obtained by microspore culture. For cytological experiments, sample sizes varied with the number of score-able cells in each experiment, but in all cases exceeded 15 cells per genotype.
Data exclusions	Data exclusion concerned several qPCR measurements for which one of the technical replicates was a clear outlier (abnormal curve or a difference of more than one cycle with other replicates). These are the only cases when measurement was discarded.
Replication	Whenever possible, replication was used. For example, we used two distinct mutant alleles for BnaC.MSH4 but only one for BnaA.MSH4 because we failed to find another null allele for that gene (we tested several misense mutations that all proved to encoded functional MSH4). The reproducibility of the mutant msh4 phenotypes was verified by the consistent observations using different plants with the same genotype (when available). The consistency between all the pyrosequencing experiments along genotypes and with previously published data supports the reproducibility of these results. The use of technical and biological individuals argues for the reproducibility of our qPCR results.
Randomization	No randomization procedures were used for our experimental design other than for qPCR analysis. For this analysis, replicates were randomly attributed to a separate qPCR plate (used as a fixed effect in the ANOVA).
Blinding	For chiasma counting in allohaploids all samples were scored blind (i.e. without knowing the genotype).

Reporting for specific materials, systems and methods

We require information from authors about some types of materials, experimental systems and methods used in many studies. Here, indicate whether each material, system or method listed is relevant to your study. If you are not sure if a list item applies to your research, read the appropriate section before selecting a response.

Materials & experimental systems

n/a	Involved in the study	
☐	☒	Antibodies
☒	☐	Eukaryotic cell lines
☒	☐	Palaeontology
☒	☐	Animals and other organisms
☒	☐	Human research participants
☒	☐	Clinical data

Methods

n/a	Involved in the study	
☒	☐	ChIP-seq
☒	☐	Flow cytometry
☒	☐	MRI-based neuroimaging

Antibodies

Antibodies used	As stated in Methods section, anti-MLH1 and anti-HEI10 antibodies, were isolated from rabbit serum. The antibodies raised against SCC3 and REC8, from guinea pig (GP) and rat serum, respectively. Anti-ASY1 and anti-ZYP1, were obtained in GP and rabbit. The secondary antibodies used were Zenon™ Alexa Fluor™ 488 Rabbit (Catalog number: Z25302) and Alexa Fluor™ Rat 568 (Catalog number:A-11077)
Validation	All the antibodies have been validated in previous studies available in the literature (see bibliography mentioned in Methods section)
